# Arginine protects against colonic barrier injury induced by long-term peritoneal dialysis

**DOI:** 10.1038/s41598-026-51876-2

**Published:** 2026-05-16

**Authors:** Junhao Dai, Yongmei Zhang, Jiaqi He, Fangping Li, Hong Xin, Xuemei Zhang, Hui Yan, Yinhang Wang

**Affiliations:** 1 Joint Center for Translational Medicine, Shanghai Fifth People’s Hospital, Fudan University, & School of Pharmacy, East China Normal University, Shanghai, 200241 China; 2 Department of Pharmacology, School of Pharmaceutical Sciences, Fudan University, Shanghai, China; 3 Weifang No. 2 Hospital, Weifang, 261041 China; 4 Department of Forensic Toxicology, Shanghai Key Laboratory of Forensic Medicine, Academy of Forensic Science, Shanghai, China; 5 Department of Neurology, Xuanwu Hospital Capital Medical University, National Center for Neurological Disorders, Beijing, China

**Keywords:** peritoneal dialysis, colon, arginine, ASL, Arg-1, Biochemistry, Diseases, Medical research, Nephrology

## Abstract

Long-term peritoneal dialysis (PD) triggers colonic dysfunction and multiple complications, yet the underlying pathogenesis and effective therapies remain elusive. We established a long-term peritoneal dialysis fluid (PDF)-treated mice model to explore molecular alterations via untargeted gas chromatography‒mass spectrometry (GC‒MS) and the data were analysed by MetaboAnalyst 5.0. After validating features against the National Institute of Standards and Technology (NIST) library and Human Metabolome Database (HMDB) libraries, we used MetaboAnalyst 5.0 to pinpoint the key discriminating metabolites and their enriched pathways, and we found abnormal arginine metabolism was closely related to colonic dysfunction. Subsequently, liquid chromatography-tandem mass spectrometry (LC‒MS/MS) and western blotting were used to determine abnormal arginine metabolism in colonic dysfunction. Additionally, the benefits of arginine were determined in PDF-treated T84 cells and the PDF-treated mouse model, evaluated the effects by histopathological staining and western blotting. Untargeted metabolomics revealed that long-term PD disrupts multiple colonic pathways, especially the arginine pathway. LC-MS/MS confirmed a marked fall in colon arginine after long-term PDF exposure. Western blot showed the downregulation of argininosuccinatelyase (ASL) and the upregulation of arginase-1 (Arg-1). Further study found that supplementing exogenous arginine to T84 cell and to PDF-treated mice reinstated mucosal morphology, restored tight junction (TJ) proteins expression, and reversed PDF-induced epithelial-barrier injury. Long-term PD leads to the injury of colonic barrier and metabolic disturbance, especially the metabolism of arginine, and exogenous arginine supplementation rescues the colon injured by PD.

**Supplementary Information:**

The online version contains supplementary material available at 10.1038/s41598-026-51876-2.

## Introduction

Peritoneal dialysis (PD) is an important replacement therapy for patients with end-stage renal disease (ESRD). Compared to haemodialysis, PD shows many benefits with low-cost, highly tolerable while preserving residual renal function^1,2^. Currently, the global growth rate of PD is approximately 8% annually^3,4^, having a higher frequency of use than haemodialysis, and Asia-Pacific caseloads are projected to double between 2010 and 2030^2,5^. Although PD provides substantial therapeutic benefits, prolonged exposure to peritoneal dialysis fluid (PDF) is a well-recognized cause of complications such as peritoneal infection and inflammation, and has been reported to be associated with gastrointestinal symptoms, including diarrhoea, constipation, and irritable bowel syndrome^6–9^. Among these, PD-induced colonic dysfunction remains mechanistically understudied.

The colon is a vital digestive organ that synthesize vitamins, propels faecal formation, and excretion^10^. Its epithelial cells form an immune barrier that seals the lumen, blocking harmful substances and microorganisms from entering the body^11–13^. Chronic PDF exposure characterized by high glucose and hyperosmotic stress leads to epithelial-barrier injury^14^. Whereas PDF-driven metabolic alteration has been dissected exhaustively in peritoneal mesothelial cells, its impact on the intestinal epithelium remains largely unexplored^15^. Studies of PD-related gut injury have largely centred on the therapeutic actions of natural or conventional drugs, leaving the intrinsic pathophysiology by which peritoneal dialysis fluids compromise intestinal integrity still poorly mapped^14,16^.

In prior research, we pinpointed the basis of PDF-evoked epithelial damage, by comparing PDF-treated mice with PDF-induced uremic mice and found that PDF alone is enough to induce injury^17^. To determine the mechanisms underlying chronic PDF-driven colonic dysfunction we first performed untargeted GC-MS on long-term PD exposure mouse models to investigate the metabolic changes of the colonic tissue. Analysis of the metabolic pathways by database comparison and Western blot was performed. We established PD-induced colonic injury models in T84 cells and C57BL/6 mice. Histological staining was used to evaluate tissue injury in vivo, wound-healing assays assessed epithelial repair in vitro, and Western blotting was performed in both models to explore the underlying molecular mechanisms. Thus, we sought to map the metabolic derangements that accompany chronic PD and to translate these insights into a targeted therapy for PD-associated colonic injury.

## Results

### PDF induces abnormalities of metabolic profiles in colons

**Fig. 1:**
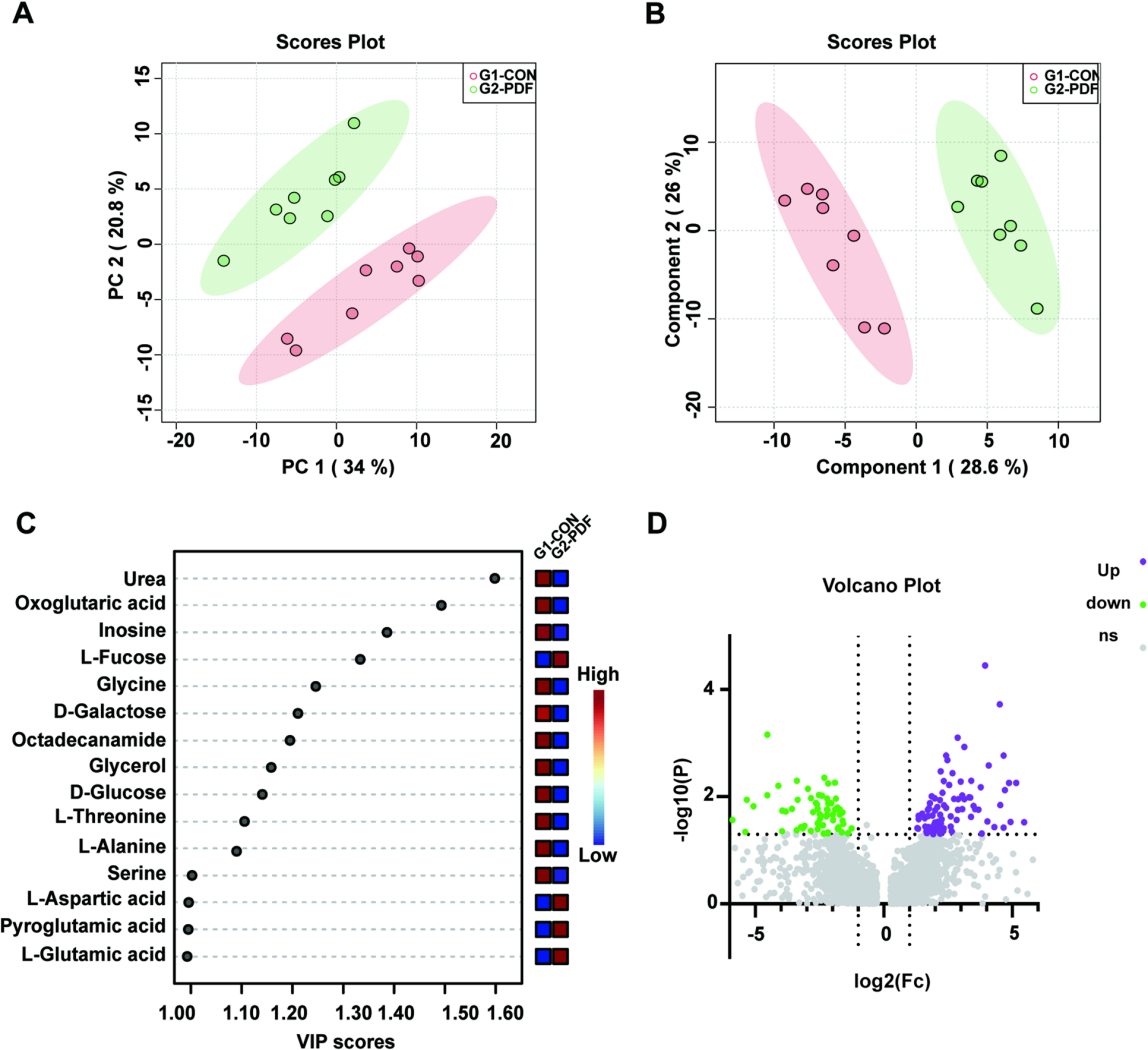
PDF treatment changes the metabolic profiles in colons. (**A**) PCA and (**B**) PLS-DA analysis score scatter plots for metabolic profiles of the control group (red dots) and PDF group (green dots) shows discrimination between the two groups. (**C**) Fifteen variables with VIP ≥ 1 screened out by PLS-DA. (**D**) Volcano plots of the most significant metabolite changes compared between two groups. Purple represents increased arginine content and green indicates decreased content of arginine in PDF mice, comparing to the control.

**Fig. 2:**
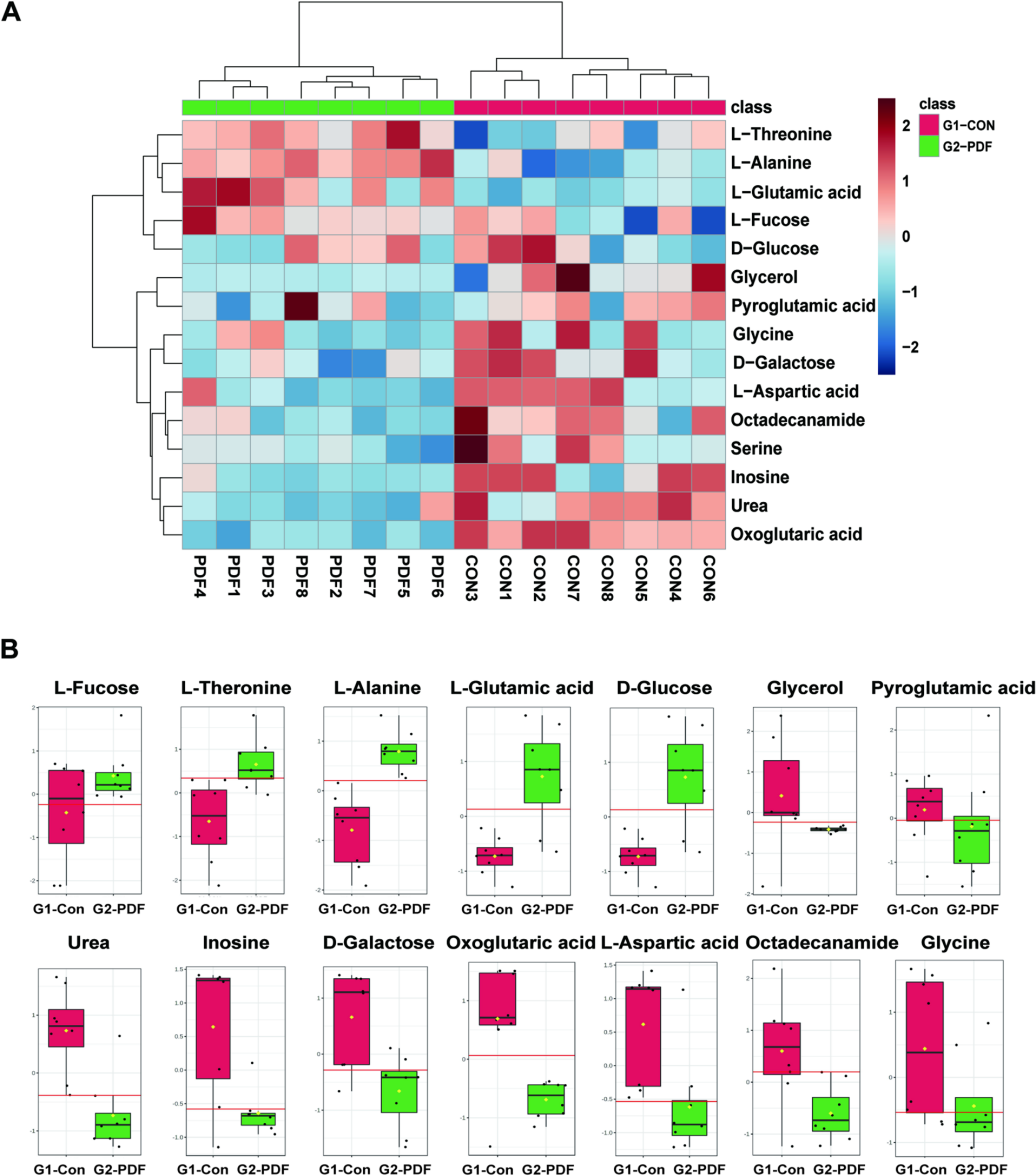
Metabolites differed markedly between the control and PDF groups. (**A**) Heatmap of representative metabolites of colon tissues between the two groups. Warmer color indicates higher concentration. (**B**) Boxplots of the fourteen most significant metabolites (*P*<0.05) in the analysis of variance results comparing the two groups, red boxes represent the control group and the green boxes represent the PDF group, *n* = 8 per group.

To study the influence of PDF on colon, we first investigated changes in the metabolic profiles of the colon. We adopted untargeted GC‒MS to analyse metabolic profiles in the colons of wildtype mice and PDF-treated mice, the results in PCA score plots are shown in Fig. 1A. PCA showed clear separation between control and PDF-treated colon samples, indicating distinct metabolic profiles. PLS-DA, a supervised pattern recognition discriminating sample with predefined class labels, showed a distinct separation trend between the two groups, along with PC1 (R2 = 0.901, Q2 = 0.775) and PC2 (R2 = 0.966, Q2 = 0.897) (Fig. 1B and Supplementary Table 1). There were 15 variables with VIP ≥ 1 identified by the PLS-DA results were identified and listed in Fig. 1C. Volcano plots found that there were variables that had *P* < 0.05 and fold change (FC) values that were ≥ 1.2 (Fig. 1D). The discriminative features were identified per the criteria of VIP ≥ 1, *P* < 0.05, and -‍1.2 ≤ FC ≤ 1.2, and 66 differential variables were screened out (Supplementary Table 2). Finally, the differences in metabolites abundance were detected and confirmed by the NIST database and Human Metabolome Database (HMDB) and then analysed through MetaboAnalyst 5.0. We found that the expression levels of some metabolites after PDF treated were significantly downregulated, including oxoglutaric acid, urea, aspartic acid, inosine, glycerol, galactose, glycine, serine, pyroglutamic acid and octadecanamide, while the expression levels of fucose, alanine, threonine, glucose, and glutamic acid, were significantly upregulated in the PDF group compared to the control group (Fig. 2A and B).

### Long-term treatment of PDF significantly decreased arginine in the colon

**Fig. 3:**
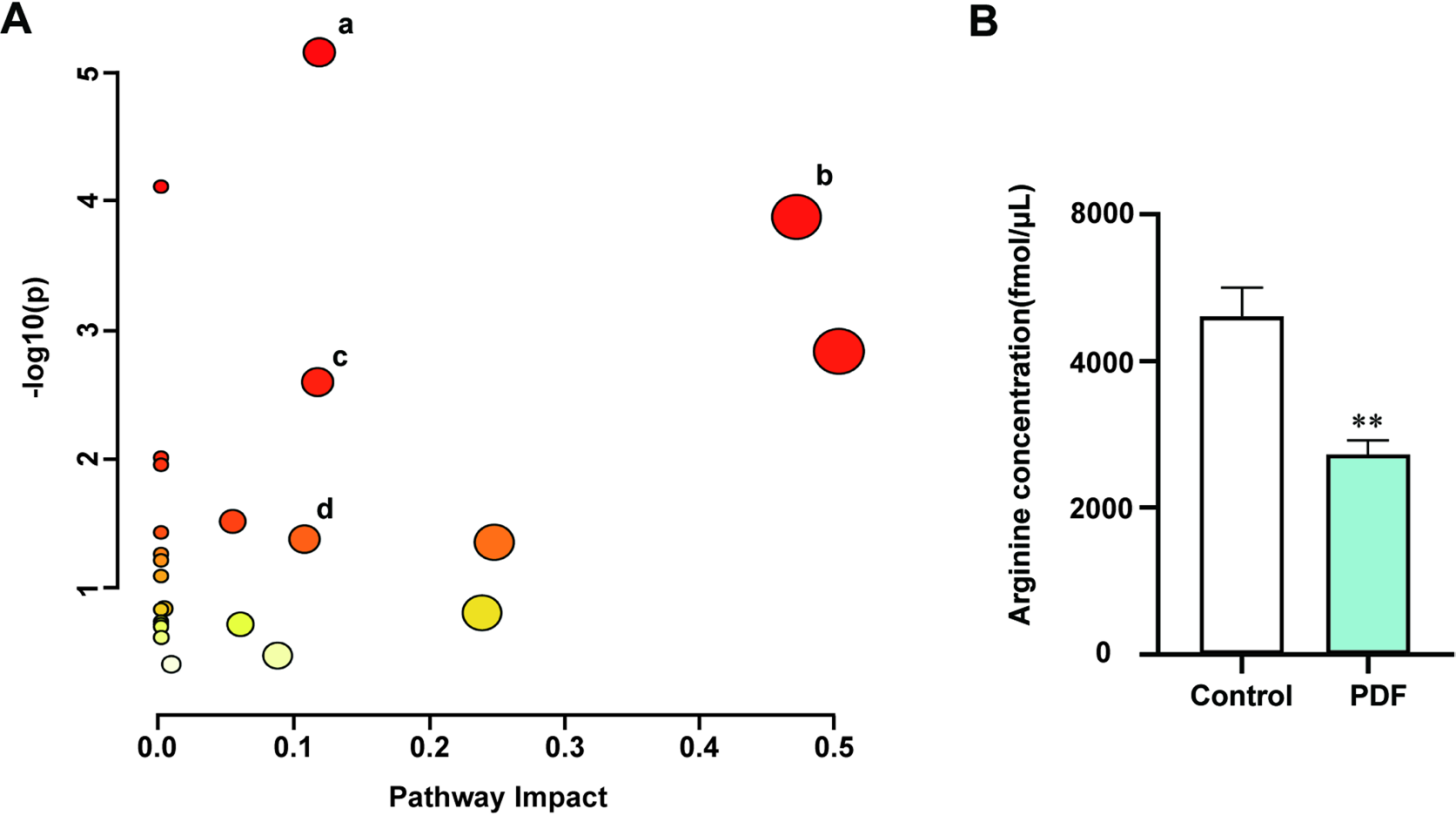
long-term treatment with PDF caused abnormal metabolic pathway and arginine deficiency in mice. (**A**) Pathway-impact map of the PDF-induced metabolome. Circle size reflects pathway-impact value; colour denotes significance based on P-values, *n* = 8 per group (a: Arginine biosynthesis, b: Alanine, aspartate, and glutamate metabolism, c: Glutathione metabolism, d: Arginine and proline metabolism). (**B**) The content of arginine in colon tissue comparing in control and PDF group. Data are presented as means ± SEM of three independent experiments, *n* = 6 per group, ^**^P<0.01 versus the control group.

In order to further identify the metabolites that are susceptible to PDF, we employed MetaboAnalyst 5.0 to perform additional analysis based on the biological significance of metabolite abundance. The HMDB IDs of the metabolites were entered into MetaboAnalyst 5.0 platform for pathway enrichment analysis. As shown in Fig. 3A, pathway analysis revealed significantabnormalities in several pathways, including arginine biosynthesis; arginine and proline metabolism; alanine, aspartate and glutamate metabolism, and glutathione metabolism. Combined with prior literature research, we determined the relationships between these pathways and colonic metabolism in sequence, and found that arginine biosynthesis and metabolism play important roles in intestinal stability^18,19^. To determine whether the change of arginine levels in the colon correlates with long-term treatment of PDF, we quantified the arginine content in the colon tissues by LC‒MS/MS. As shown in Fig. 3B and Supplementary Table 3, the concentration of arginine was significantly decreased in the PDF-treated group compared with the control group.

### PDF reduced colonic arginine by up-regulating its catabolism while down-regulating its anabolism

**Fig. 4:**
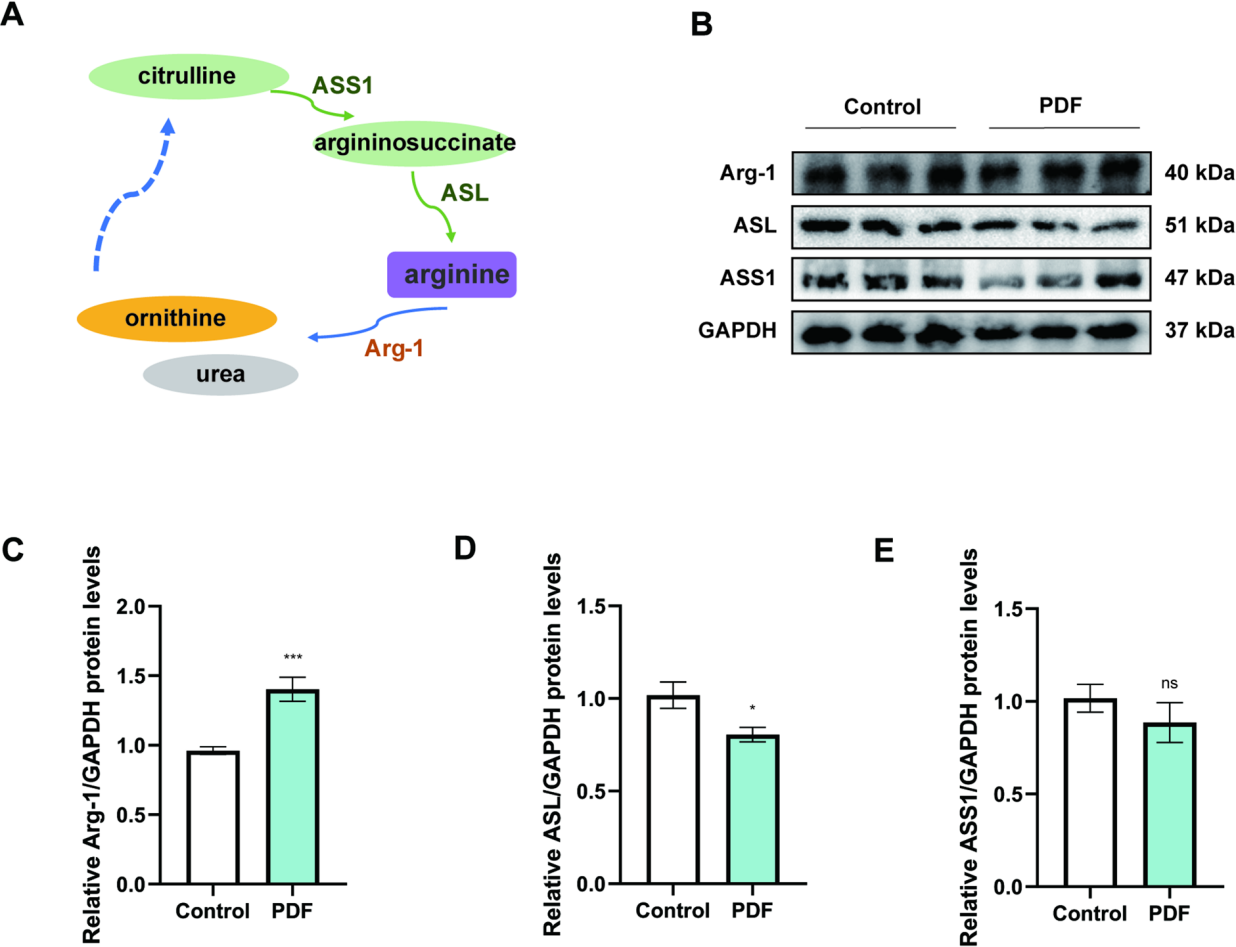
long-term PDF treatment increased Arg-1 expression and reduced the expression of ASL and ASS1 in mice colons. (**A**) Key enzymes and metabolites involved in arginine synthesis and catabolism. (**B**) The expression of Arg-1, ASL and ASS1 of colon tissues determined by western-blot, and quantitative analysis of (**C**) Arg-1, (**D**) ASL, and (**E**) ASS1 expression levels comparing of control and PDF treated mice. Data are presented as means ± SEM of three independent experiments, *n* = 6 per group, ^*^P<0.05, ^***^P<0.01 versus the control group, ns indicates no significant difference.

To determine the mechanisms underlying arginine deficiency in the colon of PDF-treated mice, we detected changes of enzymes related to arginine synthesis and metabolism in mice. Previous studies have showed that ASS1, ASL, and Arg-1 are key enzymes of arginine synthesis and metabolism in the colon (Fig. 4A)^20^. Then, we investigated the reasons for the reduction of arginine. As shown in Fig. 4B to E, the protein content of ASL and ASS1 was decreased after PDF treatment, and the decrease in ASL content remained statistically significant. Furthermore, we found that the expression of Arg-1 protein was significantly upregulated after long-term PDF treatment. Thus, reduced anabolism and increased catabolism may contribute to the arginine deficiency during PD.

### Arginine alleviate PDF induced colon injury

**Fig. 5:**
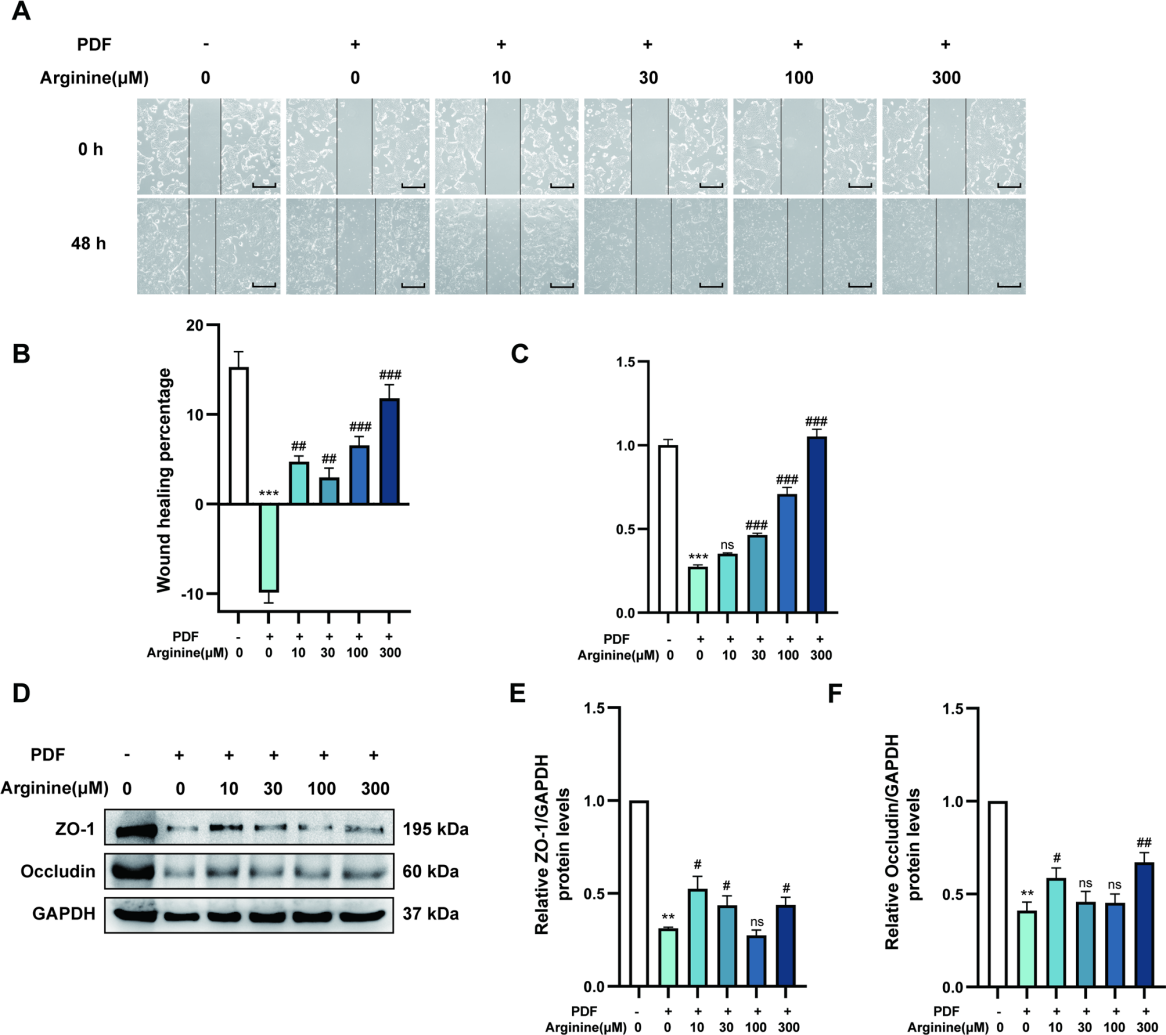
Arginine alleviated the injury induced by PDF in T84 cells. (**A** and **B**) Scratch assay of PDF-treated T84 cells incubated with 10, 30, 100 and 300 µM arginine for 48 h. Scale bars: 200 μm. (**C**) Cell viability of PDF-treated T84 cells. Cells were pre-treated with PDF and subsequently incubated with increasing concentrations of arginine for 24 h. Cell viability was assessed using the CCK-8 assay, *n* = 3. (**D**, **E** and **F**) The protein expression of ZO-1 and Occludin in PDF-treated T84 cells with different concentration of arginine supplementation by western-blot analysis. Data are presented as means ± SEM of three independent experiments, *n* = 6 per group, ^*^P<0.05, ^**^P<0.01, ^***^P<0.001 versus the control group, ^#^*P*<0.05, ^##^*P*<0.01, ^###^*P*<0.001 versus the PDF group, ns indicates no significant difference.

To further explore the effects of arginine on PDF-induced colon injury. We first evaluated the effects of arginine in PDF-treated T84 cells in vitro. T84 cells are commonly used in colonic injury studies due to their ability to form tight junction (TJ) mimicking the epithelial barrier function of the human colon^21^. As shown in Fig. 5A, B and C, PDF exposure suppressed the viability and migration of T84 cells, while, arginine treatment reversed this effect and facilitated cell migration in a dose-dependent manner, promoting the repair of the damaged intestinal epithelial barrier. The TJ proteins constitute the intestinal epithelial barrier, which is important for regulating the entrance and exit of molecules and maintaining intestinal haemostasis. Treatment with PDF markedly reduced tight junction levels of ZO-1 and occludin, whereas arginine effectively preserved TJ protein expressions (Fig. 5D, E and F).

**Fig. 6:**
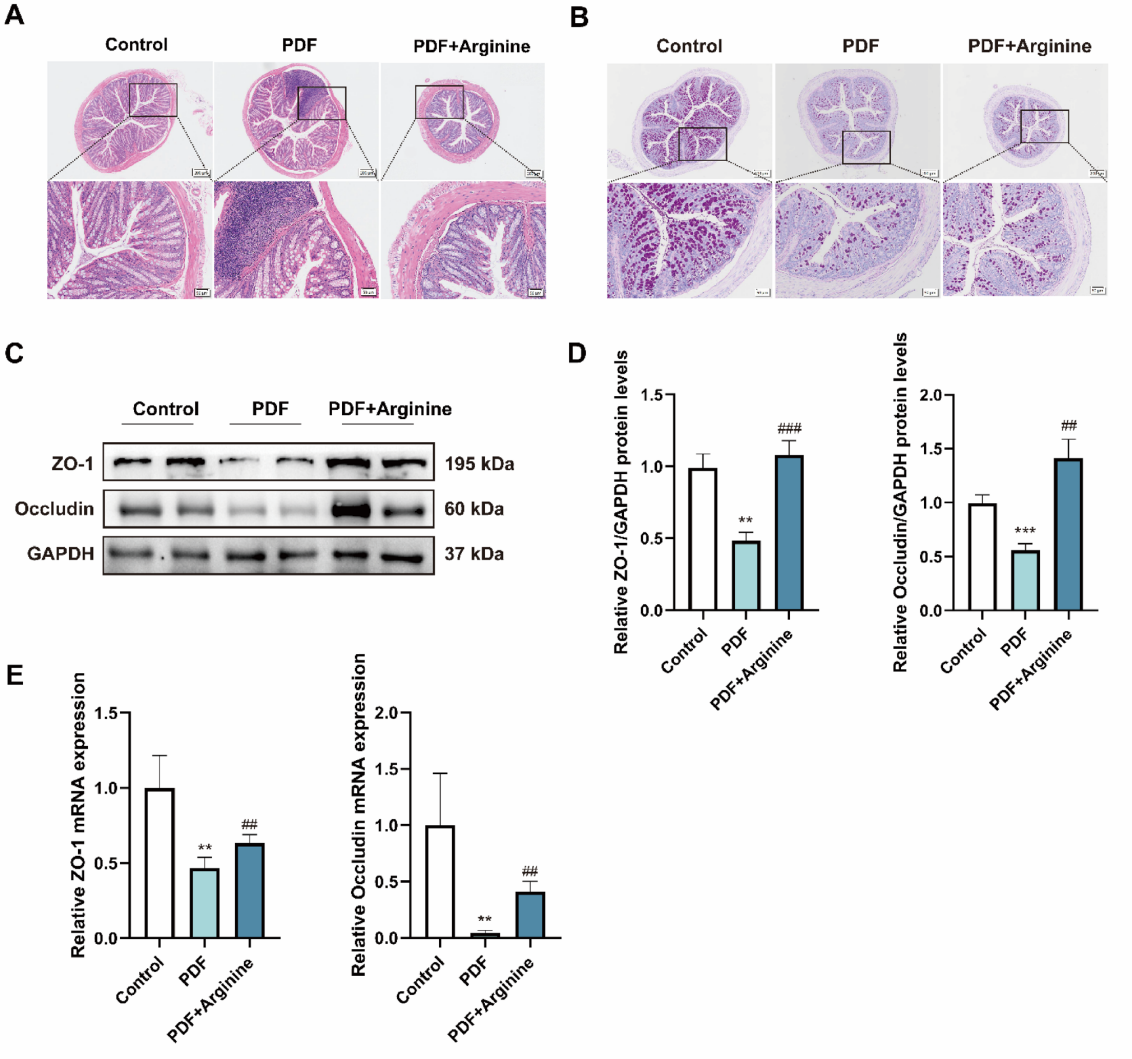
Arginine protected colons against PDF-induced injury. (**A**) Representative hematoxylin and eosin (H&E) staining of colonic sections for the appearance of the intestinal mucous membranes. Scale bars: main images, 200 μm; insets, 50 μm. (**B**) Representative periodic acid-Schiff (PAS) staining of colonic sections for indicating goblet cell morphology. Scale bars: main images, 200 μm; insets, 50 μm. (**C** and **D**) The protein expression of ZO-1 and Occludin of colon tissues revealed by western-blot analysis. (**E**) The mRNA expression of ZO-1 and Occludin of colon sections determined by RT-PCR. Data are presented as means ± SEM of three independent experiments, *n* = 6 per group, ^*^P<0.05, ^**^P<0.01, ^***^P<0.01 versus the control group, ^#^*P*<0.05, ^##^*P*<0.01, ^###^*P*<0.001 versus the PDF group.

In order to evaluate the protective effects of arginine in PD mouse model, we treated PD mice with arginine for 6 weeks. H&E staining revealed that arginine supplementation decreased pathological damage in PD mice colon, such as decreasing in inflammatory cell infiltration, defects, and irregular arrangement of crypts (Fig. 6A). Periodic acid-Schiff (PAS) staining results also showed that the phenotypes of atrophy such as decreases in goblet cell numbers and mucin secretion in the colon tissue in PD mice were reversed significantly by supplementing arginine (Fig. 6B). Finally, we performed western blotting and RT-PCR to assess the protein and gene expression of TJ proteins. Notably, chronic PDF exposure markedly inhibited ZO-1 and occludin expression; arginine supplementation obviously restored both tight-junction proteins to control level (Fig. 6C and D). The same variation trends were observed in the mRNA levels via RT-PCR (Fig. 6E). Taken together, these results indicate that arginine can effectively alleviate PDF induced colon damage both in vivo and in vitro.

## Discussion

**Fig. 7:**
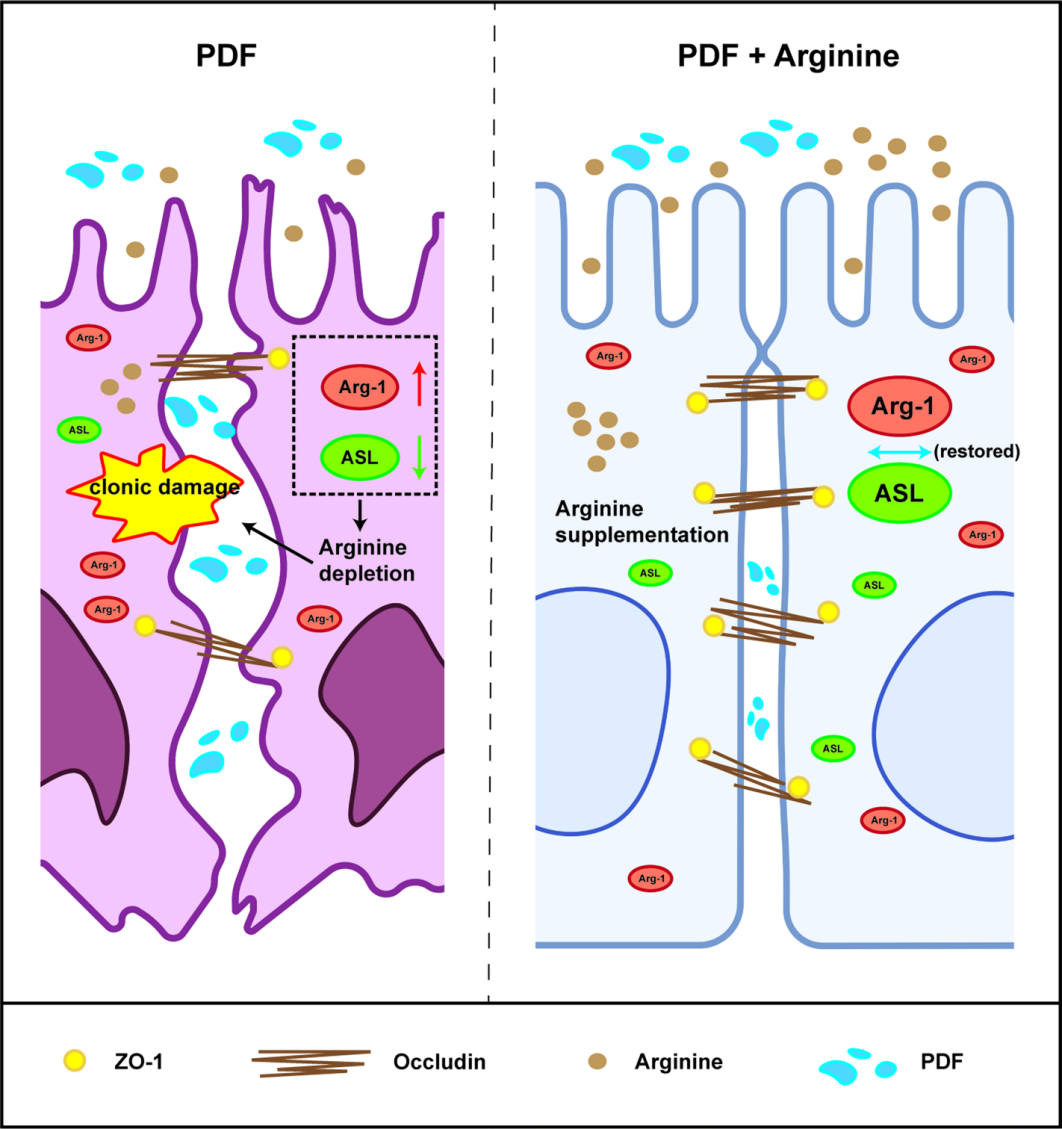
Mechanistic illustration of arginine deficiency–mediated colonic injury and the protective effect of arginine. Long-term exposure to PDF led to arginine deficiency in colonic tissues, characterized by decreased expression of the key synthetic enzymes ASL, together with increased expression of the catabolic enzyme Arg-1. The PDF treatment also disrupted epithelial tight junction integrity and aggravated mucosal injury. Supplementation with arginine restored intracellular arginine homeostasis, enhanced the expression of tight junction proteins ZO-1 and occludin, and significantly alleviated intestinal epithelial barrier damage in both in vitro and in vivo models.

Long-term PD disrupts colonic integrity and physiological function, contributing to poor prognosis. Untargeted metabolomics revealed that prolonged PDF exposure perturbs several metabolic pathways, particularly arginine metabolism. LC–MS/MS further confirmed a significant reduction of arginine levels in the colon following long-term PDF treatment. Mechanistically, PDF induced colonic dysfunction characterized by impaired arginine metabolism and decreased tight-junction protein expression. Notably, exogenous arginine supplementation in T84 cells and mice restored mucosal architecture and increased tight-junction protein levels, thereby alleviating PDF-induced epithelial barrier damage (Fig. 7). These findings suggest that arginine supplementation may represent a potential strategy to mitigate PD-associated colonic injury, which warrants further preclinical and clinical investigation.

In clinical settings, long-term PD has been reported to cause complications, including constipation, diarrhoea, and enterogenic peritonitis^22,23^. These symptoms can inconvenience patients and restrict the clinical application of PD. Unfortunately, the mechanism of PDF-related colonic complications remains unclear, and effective treatments are urgently needed. Although uraemia can trigger intestinal dysfunction, we found that long-term PD exacerbated colonic damage in a previous study^17^. Additionally, long-term PD has been shown to weaken the colonic epithelial barrier in normal mice. Herein, we explored the mechanism by which PD, a single factor, affects the colonic epithelial barrier.

Untargeted metabolomics enables high-throughput discovery and mapping of differential metabolites, linking altered pathways to their biological relevance^24–26^. Employing a metabolomic platform, we found that long-term PD resulted in abnormal biosynthesis and metabolism of arginine. LC-MS/MS revealed that the arginine content significantly decreased in long-term PDF-treated mice, thereby confirming the findings from untargeted metabolomics. Arginine is beneficial for gut microbiota homeostasis, for example, diversity of the intestinal microbiota was significantly enhanced in mice with the high arginine diet^27^. In terms of immunity, arginine is helpful for promoting innate immune activation in intestines, increasing the ability to clear pathogens, and regulating intestinal infection, and intestinal defence responses^28,29^. Arginine also plays a regulatory role in the expression of TJ related proteins and the intestinal mucosal epithelial barrier^30,31^. Here, we administered arginine to PDF-treated mice and found that it significantly alleviated PDF-related colon damage. These results may provide some references for the dietary needs of PD patients with intestinal complications. Currently the most commonly marketed PDFs are dextrose-and amino acid based, which have well documented drawbacks underscoring an urgent need for more biocompatible formulations. Recent clinical trials have shown that supplementing PDF with the parenteral dipeptide alanyl-glutamine can bolster the patient’s defense and dampens inflammation^32^. Based on our results, it is a promising strategy to add arginine analogues in dextrose-containing PDF to alleviate colon injury.

In this study, we also explored the metabolism enzymes related to synthesis and catabolism of arginine. We found a significant decrease in the expression of ASL while there was a noticeable increase in the expression of Arg-1 protein, suggesting a potential shift toward inhibited arginine synthesis and enhanced catabolism. The level of ASS1 protein also decreased, albeit not significantly like ASL, This may be particularly relevant as ASL catalyzes the final step of *de novo*arginine synthesis, and its downregulation could be a key factor associated with the observed arginine deficiency. These protein-level alterations are consistent with the observed reduction in arginine concentration, potentially reflecting disruptions in the urea cycle enzymes that govern arginine homeostasis. Previous studies have demonstrated that arginine supplementation of WT mice promotes the expansion of an antiinflammatory intestinal microbiota and alters the TLR expression pattern and the cytokine (receptor) profile^33^.. However, whether alleviating PDF-associated gut complications can be achieved by targeting Arg-1 expression warrants deeper investigation. In addition, Stettner et al. demonstrated that boosting enterocyte ASL with arginine or citrulline supplementation drives endogenous NO production, thereby restoring epithelial integrity and ameliorating colitis^18^. These results reinforce our hypothesis that arginine is protective in PDF-driven gut injury. In the future, we will dissect how PDF disrupts intestinal homeostasis through concurrent derangement of ASL and Arg1, laying the mechanistic groundwork for precision therapy.

## Methods

### Animals and animal models

Female C57BL/6J mice were obtained from Shanghai Slaccas Laboratory Animal Co., Ltd. (Shanghai, China). The study protocols were approved by the Institutional Animal Care and Use Committee at the Fudan University, School of Pharmaceutical Sciences. All animal experiments were conducted in accordance with the Guide for the Care and Use of Laboratory Animals and relevant institutional regulations. All animal experiments are reported in accordance with the ARRIVE guidelines.

All animals were group housed with free access to water and food in a room (22 ± 1 °C, 55 ± 5% humidity) with 12 h/12 h light/dark cycle. Sixteen 10-week-old female C57BL/6 mice weighing approximately 20 ± 1 g were randomly divided in the control group and the PDF group at a 1:1 ratio(*n* = 8 per group). Mice in the PDF group were daily treated with 2 mL of 4.25% dextrose-containing PDF for 6 weeks via intraperitoneal injection, while the control mice were treated with 2 mL of saline for 6 weeks. Then, the mice were sacrificed, and the colon sections were collected and analysed by untargeted GC‒MS.

We then randomized 10-week-old female C57BL/6 mice weighing approximately 20 ± 1 g into the control group, PDF group, or PDF + Arginine group, with at least 6 mice in each group to account for potential mortality during the modeling procedure. Mice in PDF group and PDF + Arginine group were both daily treated with 2 mL of 4.25% dextrose-containing PDF for 6 weeks, while the PDF + Arginine group was supplemented with 1% arginine in the drinking water. The control mice were treated with 2 mL of saline for 6 weeks. After 6 weeks of treatment, all the mice were sacrificed to study the pathological changes in the colon, 6 mice per group were randomly selected for final evaluation and all statistical analyses were performed based on *n* = 6 per group.

All mice were deeply anesthetized with isoflurane (4–5%) and then euthanized by carbon dioxide (CO₂) inhalation, followed by cervical dislocation as a secondary physical method to ensure death. All procedures were performed by trained personnel in accordance with institutional animal care guidelines and the AVMA 2020 recommendations.

### Acquisition of the untargeted GC‒MS data

Collecting the Colon and preparing the GC-MS sample. Colon sample were incubated with methanol/acetonitrile (1:1) for 20 min at 4 °C, then, collecting the supernatant after centrifugation with 13,000 rpm for 15 min. To monitor the stability and repeatability of the analytical system, a pooled quality control (QC) sample was prepared by mixing equal volumes from all experimental samples. Subsequently, add 80 µL of methoxamine hydrochloride solution (15 mg/mL, prepared in pyridine, freshly prepared and used), vortex to mix, and incubated at 40 °C for 90 min for the oximation reaction. After completion of the oximation reaction, add 80 µL of BSTFA (with 1% TMCS) and 20 µL of hexane, vortex to mix, and proceed with derivatization. The samples were vortexed again and subjected to trimethylsilylation for 60 min at 70 °C. Finally, the samples were cooled to room temperature and transferred to test tubes used for untargeted GC‒MS analysis.

The metabolic profiles were acquired using a Q Exactive GC Orbitrap GC–HRMS system (Thermo Fisher Scientific, USA) equipped with a TG-5SILMS capillary column (30 m×0.25 mm×0.25 μm, Thermo Fisher Scientific, USA). The QC samples were added to equilibrate the column system at intervals of every 5 injections. The equipment conditions were as follows: helium was used as the carrier gas at a flow rate of 1.0 mL/min, the temperature of injector was maintained at 250 °C, and the ion source and transfer line temperatures were 250 °C and 280 °C, respectively. Positive polarity full scan mode was used, the flow ratio was 15:1, and the scanning range was 60–500 m/z. The mass spectrometry resolution was 60,000 (FWHM), and 1e6 ion automatic gain control was performed. The initial temperature of the instrument was maintained at 80 °C for 1 min, the temperature was increased from 80 °C to 300 °C at a rate of 10 °C/min, and the temperature was maintained at 300 °C for 5 min. The whole process costs 28 min.

### Data processing and statistical analysis

The raw data were uploaded to XCMS Online (https://xcmsonline.scripps.edu) for data preprocessing, which included noise filtering, peak alignment, extraction, and retention time correction. The raw data processing was carried out using the following parameters: general: retention time (RT), format: minutes, polarity: positive; feature detection: centWave, ppm: 40, minimum peak width: 0.6, maximum peak width: 1.4, mzdiff: 0.01, prefilter peaks: 3, prefilter intensity: 100, noise filter: 0; RT correction: method: obiwarp, profStep: 1; grouping: method: density, bw 5, mzwid: 0.025, minfrac: 0.5, minsamp:1; diffreport: statistics.threshold.pvalue: 0.01, statistics.diffReport.value: into. Next, MetaboAnalyst 5.0 (http://www.metaboanalyst.ca/)was utilized for further data processing, including data filtering, normalization and log deformation. Subsequently, principal component analysis (PCA) was adopted to analysis and the sample projections were visualized into three dimensions of the new uncorrelated variables. Partial least-squares discriminant analysis (PLS-DA) was performed to determine the variable importance in the projection (VIP) values. Significantly changed metabolites were screened based on VIP ≥ 1, *P* < 0.05, and |fold change (FC)| ≥ 1.2 between the control group and the PDF group. According to the R^2^ and Q^2^ parameters of orthogonal PLS-DA (OPLS-DA), the fitting ability and prediction ability of the model were evaluated, as well as the occurrence of overfitting (Supplementary Table 1).

The identities of metabolites were first verified by searching the National Institute of Standards and Technology (NIST) library and the Human Metabolome Database (HMDB) based on the RT (retention time), characteristic ions and MS spectra. Finally, pathway analysis was performed using MetaboAnalyst 5.0 platform to determine the biological significance of the differentially abundant metabolites. Metabolomics data acquisition and analysis were performed using coded samples, and investigators were blinded to the experimental groups until data processing was complete.

### Determination of arginine content in the colon tissue by LC/MS-MS

The colonic tissue lysate was centrifuged at 10,000 rpm for 10 min, and the supernatant was transferred to an injection bottle. Arginine levels were determined using a targeted Multiple Reaction Monitoring (MRM) approach. The data were acquired by an H-Class instrument (Waters, USA) and an API 6500 Qtrap mass spectrometer (Sciex, USA) equipped with a capillary column (2.1 × 250 mm × 5 μm). The equipment conditions were as follows: 0.1% formic acid in water and 0.1% formic acid in acetonitrile formed the mobile phase at a flow rate of 0.2 mL/min. The loading volume was 1 µL, the temperature was maintained at 500 °C, and the ion spray voltage was 5,000 V.

For absolute quantification, an external standard calibration curve was constructed using L-arginine (Sigma-Aldrich), which ensured excellent linearity across the physiological range. The limit of detection (LOD) and limit of quantification (LOQ) were determined to be 20 fmol/µL and 60 fmol/µL, respectively (based on S/N ratios of 3 and 10). After the metabolite peak areas were extracted by Skyline, the peak area ratios of arginine to the internal standard were calculated. The absolute concentrations were then determined using the linear regression equation from the calibration curve.

### Histological analysis

Colon slices was fixed with 4% paraformaldehyde and embedded in paraffin. Subsequently, the embedded colon slices were cut into 4 μm sections and stained with haematoxylin and eosin (H&E) and periodic acid-Schiff (PAS). The histological changes and degree of inflammatory cell infiltration were observed with a brightfield microscope.

### Cell culture and arginine treatment

T84 cells were obtained from the Culture Collection of the Chinese Academy of Sciences, Shanghai, China. T84 cells were cultured in DMEM/F-12 medium supplemented with 10% foetal bovine serum (FBS). When the cells grew to 60% confluence, they were starved in serum-free medium for 8–12 h. Then, 4.25% dextrose-containing PDF and different concentrations of arginine were added to the medium without osmolarity or glucose concentration control, and the cells were cultured for 48 h.

Cell viability and proliferation were assessed using an Enhanced CCK-8 Kit (C0043, Beyotime, Shanghai, China) according to the manufacturer’s instructions. Briefly, T84 cells were seeded into 96-well plates at a density of 5 × 10^3 cells/well and incubated for 24 h to allow for attachment. Following treatment with 4.25% dextrose-containing PDF and different concentrations of arginine for the 24 h, 10 µL of CCK-8 reagent was added to each well containing 100 µL of culture medium. After incubation at 37 °C for 2 h in the dark, the absorbance was measured at 450 nm using Tecan Infinite M200 PRO multimode microplate reader (Tecan Group Ltd., Männedorf, Switzerland). The cell viability was calculated as a percentage relative to the control group. All experiments were performed in triplicates.

### Western blot analysis

The protein of colon tissue and T84 cells was extracted with radioimmunoprecipitation assay (RIPA) buffer containing protease and phosphatase inhibitors, Equal amounts of protein (20–30 µg per lane) separated by SDS‒PAGE and transferred onto a polyvinylidene difluoride (PVDF) membrane (Millipore Corporation, Billerica, MA, USA). The PVDF membrane was blocked with a skim milk solution at room temperature and incubated with the corresponding primary antibodies (anti-GAPDH (Proteintech, Rosemount, IL, USA), anti-ZO-1, anti-occludin, argininosuccinate synthase 1 (ASS1) and argininosuccinate lyase (ASL) and arginase-1 (Arg-1) (CST, Danvers, MA, USA)) at 4 °C overnight. The membranes were washed and incubated with horseradish peroxidase-conjugated secondary antibodies for 2 h. Finally, specific immunoblots were detected by enhanced chemiluminescence (ECL) and visualized using the ChemiDoc™ TouchImaging System (Bio-Rad, Hercules, CA, USA). Band intensities were quantified using ImageJ software (NIH) and quantification was performed using randomized sample IDs to ensure the objectivity of the results. Full-length uncropped Western blot images are provided in the Supplementary Materials.

### Real-time PCR

Total RNA from colon tissues was extracted with TRIzol reagent (Takara, Japan) according to the manufacturer’s instructions. The RNA was reverse transcribed with Prime ScriptTM RT Master Mix (Perfect Real Time, Takara, Japan). Then, the c-DNA samples were analysed using a Bio-Rad CFX Connect Real-time PCR system with SYBR Premix Ex Taq TM (Takara, Japan) and specific primers. The relative content of RNA in the samples was calculated by the 2^−Cq^ method and compared with that of the internal reference gene GAPDH. Primer sequences used in the RT-PCR analysis are as follows. GAPDH (mouse): forward primer: AGGTCGGTGTGAACGGATTTG, reverse primer: TGTAGACCATGTAGTTGAGGTCA; Occludin (mouse): forward primer: TGAAAGTCCACCTCCTTACAGA, reverse primer: CCGGATAAAAAGAGTACGCTGG; ZO-1 (mouse): forward primer: GCTTTAGCGAACAGAAGGAGC, reverse primer TTCATTTTTCCGAGACTTCACCA.

### Statistical analysis

Statistical analyses were performed using GraphPad Prism (version 10.0). Data distribution was assessed for normality, and homogeneity of variance was evaluated before parametric tests were applied. Comparisons between two groups were performed using Student’s t-test. For comparisons among multiple groups, one-way ANOVA followed by Tukey’s post hoc test was used. Data are presented as mean ± SEM. A p value < 0.05 was considered statistically significant.

## Supplementary Information

Below is the link to the electronic supplementary material.

Supplementary Material 1

## Data Availability

The datasets used and/or analysed during the current study are available from the corresponding author on reasonable request.
