# Supplementary material for: Arginine protects against colonic barrier injury induced by long-term peritoneal dialysis
Source: Sci Rep. 2026 May 16;16:22297. doi: 10.1038/s41598-026-51876-2 (PMC13376615; doi:10.1038/s41598-026-51876-2)
Supplement: Supplementary file 1 — Supplementary Material 1 [file 41598_2026_51876_MOESM1_ESM.pdf]

Original western blots

Figure 4B

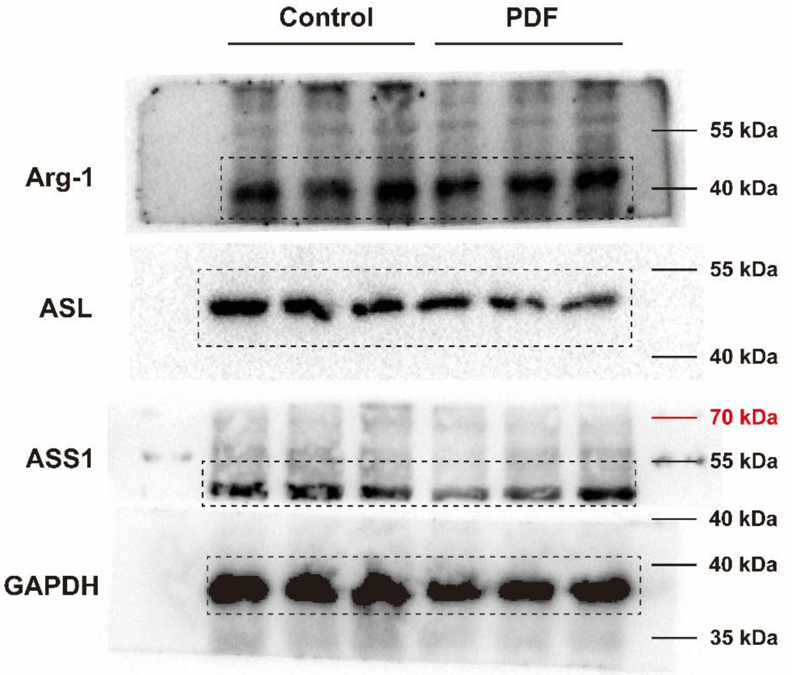

Figure 5C

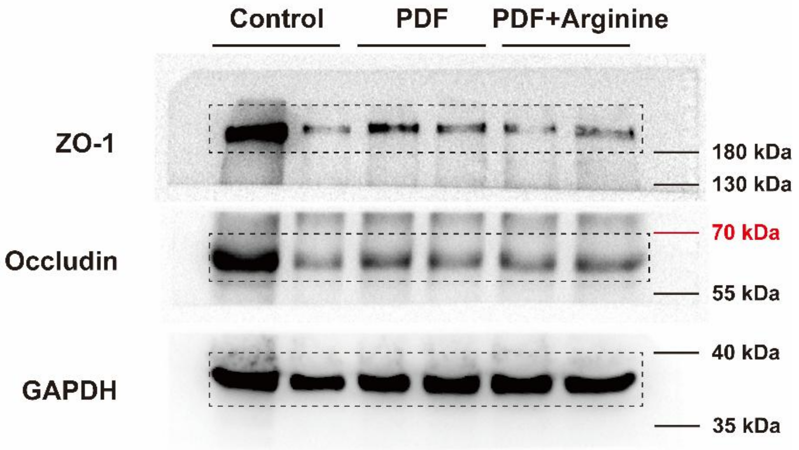

Figure 6C

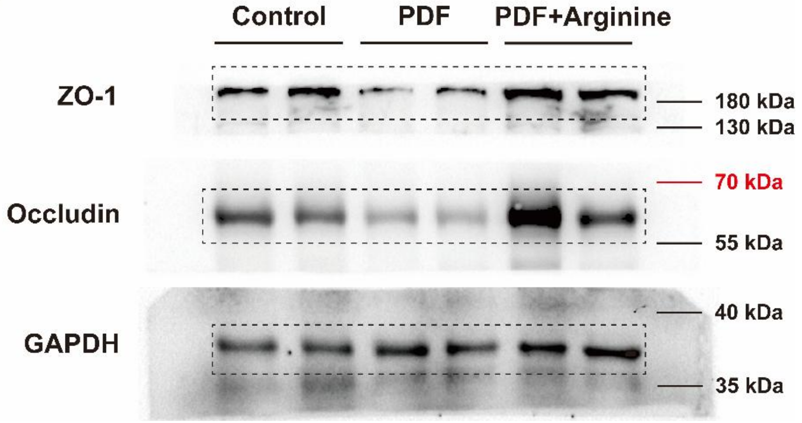

Figure S1. Original western blot images corresponding to Figures 4B, 5C, and 6C. Uncropped original western blot images are shown to indicate the source data for the bands presented in the main figures. The dashed boxes indicate the specific regions that were cropped and displayed in Figure 4B (Arg-1, ASL, ASS1, and GAPDH), Figure 5C (ZO-1, Occludin, and GAPDH), and Figure 6C (ZO-1, Occludin, and GAPDH), respectively. For Figure 4B, the blots of ASS1 and GAPDH were derived from the same membrane, whereas the blots of Arg-1 and ASL were obtained from different membranes due to molecular weight overlap, with all experiments performed under identical experimental conditions. In contrast, for Figures 5C and 6C, all blots were derived from the same original membrane. Molecular weight markers (kDa) are indicated on the right.

Supplementary Table 1. PLS-DA cross validation details

Measure	1 comps	2 comps	3 comps	4 comps	5 comps
Accuracy	0.9735	1.0	1.0	1.0	1.0
R2	0.90132	0.9662	0.99289	0.99758	0.99885
Q2	0.77507	0.89742	0.8997	0.89735	0.89132

Supplementary Table 2. Differential variables with p value, VIP and fold change

	p.value	VIP	Fc
M258T33_2	3.83E-05	1.7023	3.8052
M102T9_6	0.000388	1.5676	-4.0774
M265T22_2	0.001214	1.4787	-4.4035
M161T36_2	0.001742	1.4465	-2.182
M247T33_3	0.002031	1.4321	4.232
M248T33_1	0.003093	1.3905	4.5486
M149T32_1	0.003443	1.3794	-3.8617
M96T29_3	0.004267	1.3564	-4.7366
M83T33_2	0.004454	1.3517	-3.2224
M118T29_2	0.004466	1.3514	-2.6025
M358T20	0.004991	1.339	3.685
M171T17_3	0.008151	1.2807	2.3178
M185T36_3	0.008262	1.279	-3.8226
M171T16_4	0.009779	1.2575	2.3913
M161T32_5	0.010288	1.2509	-1.7125
M171T7_5	0.010556	1.2475	-3.4897
M204T27_2	0.010815	1.2443	2.1638
M298T11_1	0.011504	1.2361	1.3351
M311T20	0.011693	1.2339	3.3081
M295T11_3	0.011715	1.2337	-4.951
M290T33_2	0.011901	1.2315	2.1963
M152T32_4	0.011986	1.2306	-3.6105
M143T23_3	0.012121	1.229	-4.0232
M174T6_6	0.014392	1.2053	-2.4695
M294T27	0.01478	1.2015	1.8971
M182T31_1	0.015028	1.1992	-3.3653
M57T14_1	0.015991	1.1903	-1.2485
M215T17_3	0.016131	1.189	-4.703
M419T33_2	0.016404	1.1866	4.5687

M102T16_3	0.016492	1.1858	-1.9726
M195T20_3	0.017603	1.1763	2.2698
M298T12_2	0.018951	1.1653	2.6225
M332T23_1	0.019654	1.1598	1.6257
M210T10_3	0.019905	1.1579	-3.4127
M140T27	0.022062	1.1422	-3.114
M124T11_2	0.022622	1.1383	-3.3082
M208T23_2	0.022699	1.1377	-3.1215
M245T11_2	0.023969	1.1292	-2.8098
M176T11_2	0.024918	1.123	-4.3606
M153T7_3	0.02663	1.1123	-2.544
M249T8_2	0.027882	1.1049	4.8465
M114T8_7	0.02797	1.1044	-1.5316
M156T12_5	0.028059	1.1038	-3.6076
M140T7_1	0.02866	1.1003	-3.9359
M151T20_1	0.029311	1.0966	-1.8246
M160T33_5	0.030294	1.0912	-2.4118
M146T22_3	0.030784	1.0885	1.4566
M132T29_3	0.032283	1.0805	-3.8133
M141T20_1	0.032402	1.0798	1.3379
M208T12	0.033668	1.0733	-2.5372
M85T15_4	0.034212	1.0705	1.9534
M57T13_2	0.034491	1.0692	-1.0453
M265T10	0.035058	1.0663	-3.1455
M177T33_3	0.035484	1.0642	-2.8427
M283T18_2	0.035939	1.062	4.5876
M181T20	0.036367	1.06	-2.9473
M99T31_2	0.036372	1.0599	-3.567
M171T17_1	0.042957	1.0303	1.9823
M211T20	0.043058	1.0299	-2.702
M192T35_3	0.043292	1.0289	1.4511
M157T30_1	0.043692	1.0272	3.9783
M140T12_3	0.044365	1.0244	-3.3739
M156T12_4	0.04468	1.0231	-3.6236
M367T37_2	0.045965	1.0179	-2.5143
M76T15_3	0.048862	1.0065	3.1786
M186T10_1	0.049721	1.0032	-2.5334

Supplementary Table 3. The content of arginine in the colon detected by LC-MS

Number	Concentration（fmol/μL）

Con-1	4621.884
Con-2	4995.905
Con-3	4205.206
PDF-1	2721.067
PDF-2	2924.752
PDF-3	2542.601
